# Supplementary material for: Using Whole Slide Gray Value Map to Predict HER2 Expression and FISH Status in Breast Cancer
Source: Cancers (Basel). 2022 Dec 17;14(24):6233. doi: 10.3390/cancers14246233 (PMC9777488; doi:10.3390/cancers14246233)
Supplement: Supplementary file 1 [file cancers-14-06233-s001.zip › cancers-2056280-supplementary.pdf]

cancers

MDPI

Supplementary Materials

Using Whole Slide Gray Value Map to Predict HER2 Expression and FISH Status in Breast Cancer

Table S1. HER2 IHC classification performance of GrayMap methods by cross-validation classification in subgroup of 0/1+, 2+ and 3+.

	Fold	precision	recall	f1-score	support
0/1+	1	1	0.333	0.5	3
	2	1	0.75	0.857	8
	3	1	1	1	7
	4	0.667	0.4	0.5	5
	5	1	0.333	0.5	3
	Avg.	0.933	0.563	0.671	
	Std.	0.149	0.299	0.240	
2+	1	0.943	1	0.971	33
	2	0.941	1	0.97	32
	3	0.968	1	0.984	30
	4	0.906	0.967	0.935	30
	5	0.941	1	0.97	32
	Avg.	0.940	0.993	0.966	
	Std.	0.022	0.015	0.018	
3+	1	1	1	1	10
	2	1	1	1	6
	3	1	0.889	0.941	9
	4	1	1	1	10
	5	1	1	1	10
	Avg.	1.000	0.978	0.988	
	Std.	0.000	0.050	0.026	

Table S2. HER2 FISH prediction performance of GrayMap methods on subgroup of 0/1+, 2+ and 3+.

	precision	recall	f1	jaccard	accuracy	specificity
0/1+	1.000 ± 0.000	0.333 ± 0.250	0.500 ± 0.333	0.333 ± 0.250	0.941 ± 0.056	1.000 ± 0.000
2+	0.868 ± 0.072	0.836 ± 0.067	0.852 ± 0.050	0.742 ± 0.075	0.895 ± 0.034	0.928 ± 0.045
3+	1.000 ± 0.000	1.000 ± 0.000	1.000 ± 0.000	1.000 ± 0.000	1.000 ± 0.000	---
